# *Arabidopsis* cell expansion is controlled by a photothermal switch

**DOI:** 10.1038/ncomms5848

**Published:** 2014-09-26

**Authors:** Henrik Johansson, Harriet J. Jones, Julia Foreman, Joseph R. Hemsted, Kelly Stewart, Ramon Grima, Karen J. Halliday

**Affiliations:** 1 Synthetic and Systems Biology (SynthSys), University of Edinburgh, CH Waddington Building, Mayfield Road, Edinburgh EH9 3JD, UK

## Abstract

In *Arabidopsis*, the seedling hypocotyl has emerged as an exemplar model system to study light and temperature control of cell expansion. Light sensitivity of this organ is epitomized in the fluence rate response where suppression of hypocotyl elongation increases incrementally with light intensity. This finely calibrated response is controlled by the photoreceptor, phytochrome B, through the deactivation and proteolytic destruction of phytochrome-interacting factors (PIFs). Here we show that this classical light response is strictly temperature dependent: a shift in temperature induces a dramatic reversal of response from inhibition to promotion of hypocotyl elongation by light. Applying an integrated experimental and mathematical modelling approach, we show how light and temperature coaction in the circuitry drives a molecular switch in PIF activity and control of cell expansion. This work provides a paradigm to understand the importance of signal convergence in evoking different or non-intuitive alterations in molecular signalling.

## Abstract (web summary)

Light and temperature interact to control hypocotyl elongation in *Arabidopsis*. Here, Johansson *et al.* use experimental data and mathematical modelling to describe a photothermal molecular switch where temperature governs whether light represses or activates elongation.

## Graphical abstract

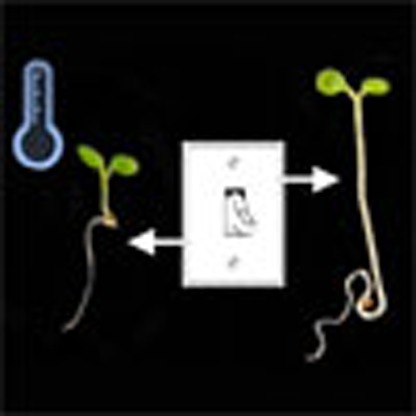

The light-triggered conversion from skotomorphogenic to photomorphogenic growth (de-etiolation) is a vital developmental step in the early plant life cycle. Seedling de-etiolation results in the opening and expansion of the cotyledons, unfolding of the apical hook and inhibition of hypocotyl elongation^1^. The previous decades of research have identified multiple photoreceptors, downstream factors and hormonal pathways regulating and fine tuning this process^2^,^3^,^4^,^5^, often utilizing seedling hypocotyl length to measure the extent of de-etiolation. As elongation of this simple organ is also regulated by ambient temperature, the hypocotyl provides a tractable system to study the intersection of light and temperature^6^,^7^,^8^,^9^,^10^,^11^.

The red light photoreceptor, phytochrome B (phyB) is exquisitely tuned to detect changes in the ambient light environment. Synthesized in its inactive Pr form, phyB is photoconverted to active Pfr following exposure to red light, while far-red light reverses this process. Pr to Pfr conversion triggers cytosolic to nuclear localization and aggregation in subnuclear foci or speckles^12^. Pfr is also subject to ‘dark reversion’, or thermal relaxation to the ground Pr state, independently of light^13^. The photoreversibility of phyB delivers dynamic light quality sensing, particularly attuned to detect far-red-rich environments that signify potential crowding from neighbouring plants. However, phyB is also a reliable sensor of light quantity. This is exemplified in the much studied fluence rate response curve, where phyB suppression of hypocotyl cell expansion increases with red light fluence rate^14^,^15^. A recent kinetic model of phyB action elucidated the molecular properties of phyB required for light intensity sensing^14^. This study showed that the photochemical reactions alone were insufficient to deliver the fluence rate response, and in fact dark reversion and nuclear speckling were important dynamic processes in this response. Modelling approaches have also been invaluable in deciphering the highly complex phyA signal transduction^16^.

Hypocotyl elongation is under the dual control of phyB and the phytochrome-interacting factors (PIFs) bHLH transcription factors. PIFs are potent promoters of hypocotyl extension, and phyB constrains PIF action by triggering PIF degradation by the proteasome and through direct sequestration preventing PIF binding to target promoters^17^,^18^. Conversely, PIFs induce constitutive photomorphogenic 1 (COP1) E3 ligase-dependent proteolysis of phyB^19^. However, we do not yet understand how this dual-negative regulation impacts signalling and the end point physiological responses.

While phyB action represses hypocotyl elongation, warm temperature promotes it. Several studies have shown that PIF4, and to a lesser extent PIF5 stimulate hypocotyl extension in response to heat^5^,^7^,^10^,^11^,^20^,^21^. Thus, PIF4 and PIF5 operate at the intersection of light and temperature signalling. High temperature promotes PIF4 binding to the promoters of genes that control biosynthesis of the plant hormone auxin, which in turn promotes elongation^7^,^8^,^11^. Interestingly, an earlier report showed that light is required for the auxin-induced hypocotyl cell elongation at high temperatures^8^. This observation is in apparent conflict with current understanding that light, acting through photoreceptors such as phyB, is a strong suppressor of hypocotyl extension.

The bZIP transcription factor long-hypocotyl 5 (HY5) acts in opposition to PIFs. This antagonism is evident in loss-of-function mutants: *hy5* has a long-hypocotyl phenotype, while *pif* mutants are shorter than wild type^22^,^23^. Like PIFs, HY5 protein levels are regulated by light, but unlike PIFs that are degraded, light promotes HY5 protein accumulation. Here light acts in part by reducing the nuclear levels of the E3 ubiquitin ligase component COP1, which targets HY5 for proteasome-mediated degradation^24^,^25^. HY5 and PIFs are known to share a broad suite of gene targets and recently they have been shown to elicit opposing action through collocation at gene promoters^26^,^27^. In common with PIF4, HY5 participates in temperature signalling but, while PIF4 operates at higher temperatures, HY5 is an important regulator of cold signalling, promoting anthocyanin accumulation^20^,^28^,^29^.

In this study, using the hypocotyl elongation response as a physiological readout, we aimed to gain a systems level understanding of how changes in the light and temperature environment impact signalling through the phyB–PIF motif. Here we report that the ‘classical’ hypocotyl response to fluence rate is temperature dependent, exposing a gap in our fundamental understanding of light signal transduction. To investigate this, we developed the first phyB signalling model that incorporates phyB–PIF dual-negative feedback regulation. Our data show that phyB action does not saturate at relatively low fluence rates, as previously thought, but continues to inhibit hypocotyl elongation in high light. At cooler temperatures, this high fluence rate response requires the action of HY5. By analysing the phyB–PIF signalling module at a systems level, we solve the dilemma of how light, which is a potent repressor of hypocotyl extension, can switch to a promoter in the warm.

## Results

### Red light promotes hypocotyl extension at high temperature

**Figure 1:**
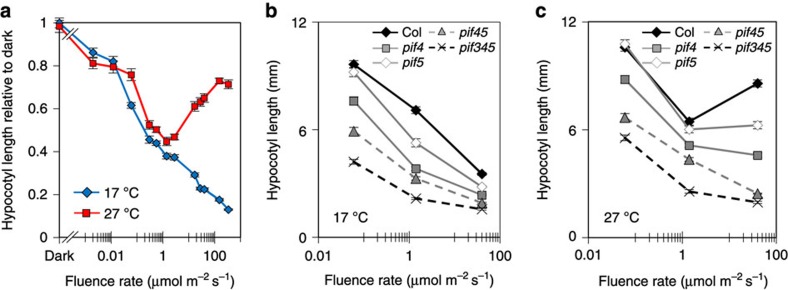
The fluence rate-dependent switch in hypocotyl regulation at warm temperatures requires PIFs. (**a**) Fluence rate response curves measuring hypocotyl elongation of 7-day-old WT seedlings grown in continuous red light at 17 °C and 27 °C shown as relative to dark at 17 °C. Sample number >17, error bars represent s.e.m. (**b**,**c**) Fluence rate response curves measuring hypocotyl length of WT, *pif4-101, pif5-3*, *pif4-101pif5-3* and *pif3-3pif4-2pif5-3* at 17 °C (**b**) and 27 °C (**c**) grown at indicated fluence rate of red light. Sample number >16, error bars represent s.e.m.

A plethora of literature supports the long-held view that hypocotyl extension is progressively suppressed by increasing red light fluence rates^30^. While we observe this standard response at 17 °C and 22 °C (Fig. 1a and Supplementary Fig. 1a), at 27 °C, above a low-light threshold (~1 μmol m^−2^ s^−1^), elevated fluence rates incrementally promote elongation. This finding holds true for multiple *Arabidopsis* accessions (Supplementary Fig. 1b) illustrating that across ecotypes heat induces a complete response inversion from light inhibition to light promotion of hypocotyl extension. Our data illustrate that light and temperature do not act in isolation; rather, the convergence of these signals drives a ‘photothermal switch’ in response.

As PIF4 and PIF5 have also been implicated in temperature-dependent hypocotyl elongation, they presented good candidate regulators of the photothermal switch^6^,^10^,^20^,^31^. In fact, sequential removal of PIF4, PIF5 and PIF3 led to a gradual suppression of hypocotyl length at both 17 °C and 27 °C and importantly, abolished the fluence rate-driven hypocotyl extension at 27 °C (Fig. 1b,c). Previous studies in white light have shown that PIF4 regulates hypocotyl extension in a temperature-dependent manner^10^,^20^. Our data demonstrate that in red light, PIF4 operates over a temperature range. Moreover, the large impact of *pif4* at both temperatures and the complete elimination of light-induced hypocotyl extension at 27 °C, indicate that PIF4 has a prominent role in the photothermal switch.

### At 27 °C hypocotyl length does not correlate with PIF levels

**Figure 2:**
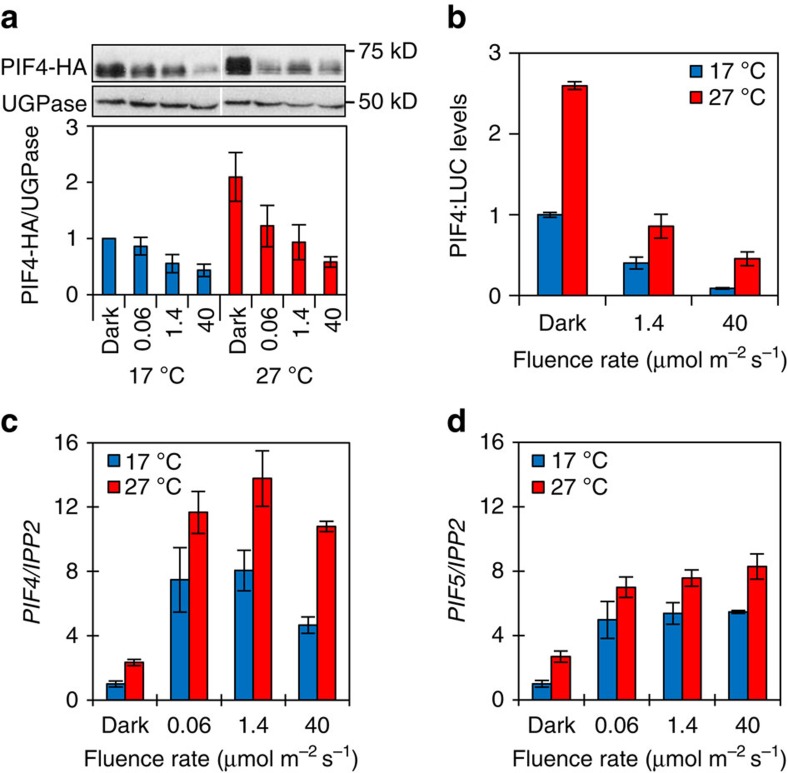
PIF protein and transcript levels do not correlate with activity. (**a**) Western blot of 6-day-old *35S::PIF4-HA* seedlings grown at indicated temperature and continuous fluence rate of red light using an HA-specific antibody for detection of PIF4-HA and anti-UGPase as loading control (upper panel). Quantification of PIF4-HA protein levels relative to UGPase (lower panel). Error bars represent s.e.m., *n*=3. (**b**) PIF4-LUC levels relative to total protein in 6-day-old seedlings grown at indicated temperatures and fluence rates of red light. Error bars represent s.e.m., *n*=3. Quantification of *PIF4* (**c**) and *PIF5* (**d**) transcript levels relative to *IPP2* in seedlings grown as in **a**. Error bars represent s.e.m., *n*=3.

As biological reactions are temperature dependent, we postulated that at 27 °C phyB signalling may become less efficient at higher fluence rates due to increased rate of photoconversion between Pr and Pfr. This scenario predicts that at 27 °C phyB would be less effective at degrading PIF proteins at increased fluence rates. However, we observe a strong fluence rate-dependent depletion of PIF4-HA, PIF4-LUC and PIF3-LUC at both temperatures (Fig. 2a,b and Supplementary Fig. 1c). It therefore appears that phyB regulation of PIF protein levels is not compromised at high temperatures. As the PIF constructs we used in this study were all driven by the 35S promoter, we wanted to establish whether control was delivered at the transcript level. While we did observe the previously reported light and temperature dependence of *PIF4* and *PIF5* transcript abundance^10^,^32^,^33^, levels did not correlate with the hypocotyl response at either 17 °C or 27 °C (Fig. 2c,d). These data suggest that phyB remains active at 27 °C and, as a consequence, PIF levels inversely correlate with fluence rate. PhyB depletion of PIF levels is thought to underlie the classical fluence rate-dependent suppression of hypocotyl elongation, yet at 27 °C, we observe the converse.

### Development of the phyB–PIF model

**Figure 3:**
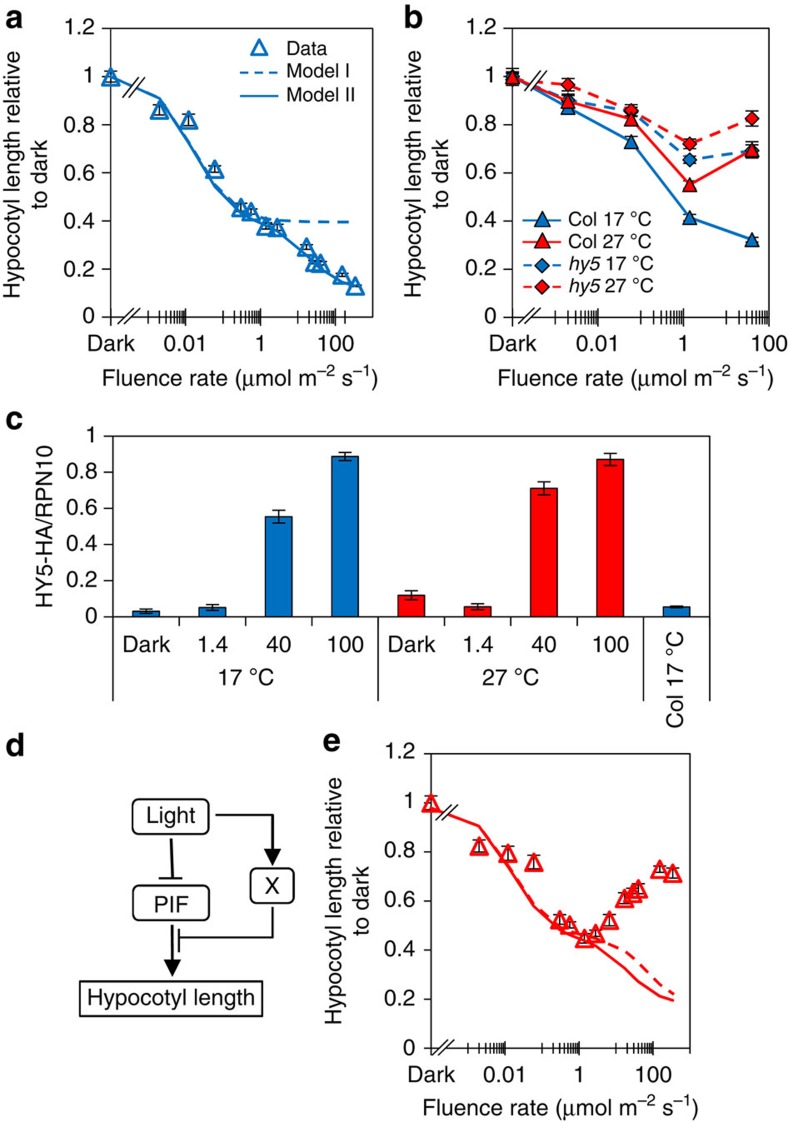
Suppression of PIF activity by X is required to repress hypocotyl elongation at high fluence rates. (**a**) Comparison of 17 °C experimental data with model I and model II simulation. (**b**) Fluence rate response curve measuring hypocotyl elongation of 7-day-old WT and *hy5* seedlings grown under continuous red light at 17 °C and 27 °C. Sample number >20, error bars represent s.e.m. (**c**) Quantification of HY5-HA protein levels relative to RPN10 in seedlings grown at indicated temperatures and fluence rates. Error bars represent s.e.m., *n*=3. (**d**) Diagram of coherent feed-forward motif at 17 °C. (**e**) Temperature dependency of Model II. Experimental data at 27 °C (red triangles), simulation data at 27 °C with temperature dependency (X, red solid line: X and all other temperature-dependent parameters, red-dashed line).

To help us understand the light- and temperature-induced changes that give rise to this altered signalling through the phyB–PIF circuitry, we adopted a mathematical modelling approach. The Rausenberger *et al.*^14^ phyB model incorporates early events of phyB signalling including dimerization, light-dependent Pr to Pfr transformation, Pfr to Pr dark reversion, translocation from cytoplasm to nucleus, formation of nuclear speckles and light-dependent degradation^13^,^14^,^19^. We extended this model to incorporate the mutual proteolytic destruction of phyB and PIF (following phyB Pfr–PIF interaction) and PIF control of hypocotyl length; we refer to this as Model I (Supplementary Fig. 2a). Similar to the Rausenberger model, Model I can simulate phyB-controlled fluence rate-dependent hypocotyl inhibition (Fig. 3a).

### The model predicts X is required for phyB action

In Model I (and the Rausenberger phyB model), the phyB-induced fluence response saturates at low fluence rates predicted to achieve maximum photoconversion to active Pfr. In contrast, our 17°C data show that the hypocotyl inhibition response continues into the higher fluence rate range (Fig. 3a). This discrepancy identifies a gap in our understanding of light control of this response. In Model I, hypocotyl elongation is directly linked to PIF levels (Supplementary Methods). Therefore, further suppression of hypocotyl elongation inhibition at higher fluence rates could in principle, be induced by a concomitant rise in phyB and fall in PIF levels. To test this, phyB and PIF3 protein (that is acutely sensitive to phyB levels) were measured over an extended fluence rate range. Our data illustrate that both proteins deplete to a stable low level at 17 °C and at 27 °C (Supplementary Figs 1c and 3a). Based on this finding, we postulated the existence of a new fluence rate component X which represses PIF activity, particularly at higher fluence rates (see Model II in Supplementary Methods; Supplementary Fig. 2c). With this model modification, we were indeed able to match the experimental data: compare dashed line (Model I), with solid line (Model II) (Fig. 3a). As we observed hypocotyl elongation rather than inhibition at 27 °C, we surmised that, as well as fluence rate dependent, X may be temperature dependent, with greater potency at cooler temperatures.

### Experimental validation of HY5 as a component of X

Several antagonists of PIF action have been reported^2^,^34^,^35^, however, in this category, the bZIP transcription factor HY5 is a good candidate for X as it is known to be regulated by both light and temperature^24^,^28^. A stipulation of Model II is that X action increases with fluence rate. Our data show that the previously reported *hy5* long-hypocotyl phenotype and the accumulation of HY5 protein are both strictly fluence rate dependent in red light (Fig. 3b,c)^22^. HY5 therefore fulfils the functional requirements for X in our model and likely constitutes a major component of X. Like HY5, HFR1 and DELLAs are well-known suppressors of PIF action, we therefore tested whether they could contribute to X^2^,^3^,^26^,^27^,^34^. In line with previous observations, we did not observe a marked *hfr1* hypocotyl phenotype in our red light conditions^36^,^37^,^38^ (Supplementary Fig. 4a). In contrast, at 17 °C and 27 °C, we did observe a subtle increase in the *della4* (*rgl1-1;rgl2-1;gai-6;rga-2)* mutant hypocotyl length compared with the wild type at the higher 40 μmol m^−2^ s^−1^ fluence rate (Supplementary Fig. 4b). This indicates that DELLAs may contribute to X, but in a more minor way. We also speculated that HY5 action may be temperature dependent. Figure 3c shows that in continuous red light, HY5 protein levels are not altered by temperature in our experimental range, but the impact of *hy5-215* is more influential at 17 °C than 27 °C, suggesting that HY5 is indeed more active at cooler temperatures (Fig. 3b). Our data therefore validate the necessity for X. It also suggests that at moderate temperatures, hypocotyl elongation is regulated by a coherent feed-forward motif where levels and activity of PIFs are repressed by light (Fig. 3d). Simulated data obtained from Model II illustrates that failure of either motif arm leads to incomplete hypocotyl inhibition (Supplementary Fig. 5).

### Development of Model III, the phyB–PIF photothermal model

To gain a more thorough understanding of how temperature impacts signalling through the phyB–PIF network, we incorporated temperature regulation into Model II by assigning Q_10_ values to specific parameters (Supplementary Methods). We observe a modest temperature-induced rise in phyB levels in dark grown seedlings (Supplementary Fig. 3a) and therefore assigned Pr formation rate, (*n*_1_) mildly temperature sensitive. Likewise, fluence rate depletion of phyB (by PIFs) and PIF4/PIF3 (by phyB) is moderately temperature dependent, identifying m_3_ (PIF-dependent degradation of Pfr) and m_4_ (Pfr-dependent degradation of PIF) as temperature parameters (Fig. 1d; Supplementary Figs 1c and 3a and Supplementary Methods). PIF4 formation rate (*n*_2_), dark reversion rate (*p*_1_) and the basal rate of cell expansion (*α* in the model), are reported to be temperature dependent^6^,^14^,^20^. Finally, our genetic data (Fig. 3b) suggested the activity of X (HY5) b_1_, is also temperature dependent. Interestingly, a detailed sensitivity analysis identified a large subset of these parameters (*n*_1_, *n*_2_, *m*_3_, *m*_4_ and *α*) as those causing, on perturbation, a significant change in light-dependent hypocotyl elongation. The analysis also identified *g*_1_ (Michaelis–Menten constant) as a further sensitive parameter (Supplementary Fig. 6; Supplementary Table 1 and Supplementary Methods).

### ‘X’ cannot generate the photothermal switch

As we have shown that X (HY5) strongly antagonizes PIF action at 17 °C, but not at 27 °C, we were intrigued to establish whether X could generate the temperature-activated switch in behaviour. The model, however, could not generate the observed fluence rate-dependent increase in hypocotyl length at 27 °C, suggesting X action does not drive the switch (Fig. 3e). A global parameter search illustrated that over an extensive range of possible Q_10_ values, Model II cannot recapitulate the switch (Supplementary Methods). These limitations of the model at 27 °C indicated that additional components were required to elicit the switch in light control of elongation.

### Light promotes PIF action at 27 °C

**Figure 4:**
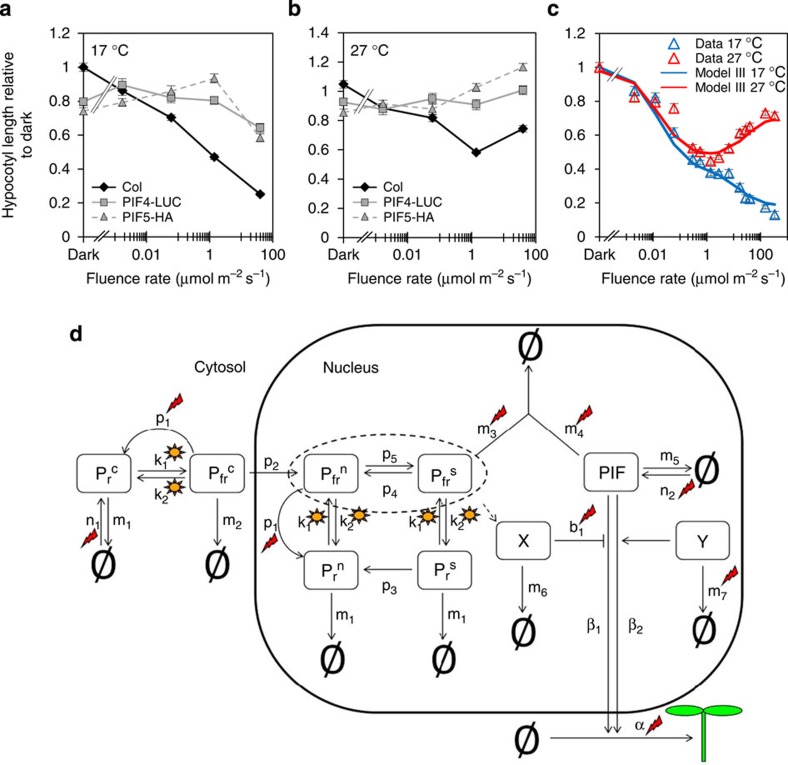
Model III predicts that light promotes PIF activity at 27 °C. (**a**,**b**) Hypocotyl measurements of 7-day-old WT, overexpressing lines of PIF4-LUC and PIF5-HA in 17 °C (**a**) and 27 °C (**b**) grown at indicated fluence rate of continuous red light and shown as relative to the dark value of WT at 17 °C. Sample number >18, error bars represents s.e.m. (**c**) Experimental data and simulated data from Model III of hypocotyl elongation at indicated fluence rates of red light and temperature. (**d**) A schematic representation of Model III including factors X and Y. X can be modelled as either phyB dependent or phyB independent. Suns and flashes represent light- and temperature-regulated events, respectively.

When we analysed data from seedlings expressing 35S:PIF4-LUC or 35S:PIF5-HA, we noted that hypocotyl elongation is suppressed at higher fluence rates at 17 °C but not at 27 °C (Fig. 4a,b). Furthermore in the 35S:PIF5-HA seedlings, we observed a fluence rate-dependent rise in hypocotyl extension. This provided additional support for the inclusion of a light-regulated PIF suppressor (X) that operates in the cool, but also suggested PIF action may be boosted by light at 27 °C. As light is thought to solely act as a negative regulator of PIFs, this proposition is at apparent odds with current understanding. To test this hypothesis, we introduced a second fluence rate-dependent component Y, which promotes PIF activity in a temperature-dependent manner (Fig. 4c,d and Model III, Supplementary Methods). With the inclusion of Y, Model III can fully replicate the photothermal switch, which supports our hypothesis, and suggests that the convergence of light and temperature on PIF activity underlies the response inversion.

**Figure 5:**
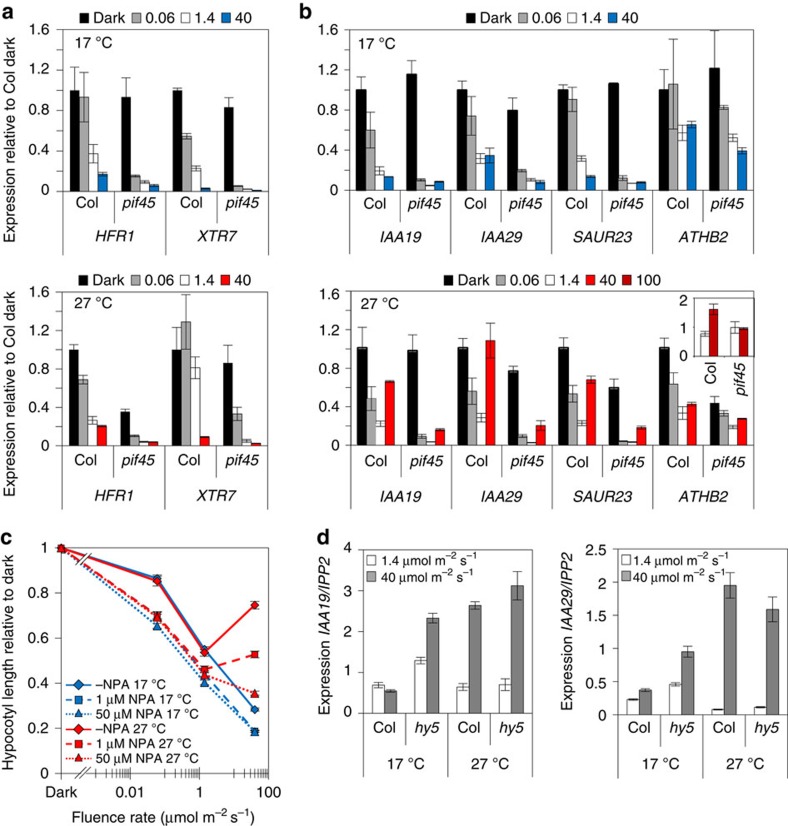
At 27 °C, PIF4/5-dependent transcriptional regulation of auxin targets is promoted at high fluence rates. (**a**,**b**) Transcript levels of *HFR1*, *XTR7*, *IAA19*, *IAA29*, *SAUR23* and *ATHB2* relative to *IPP2* in 6-day-old WT and *pif4-101pif5-3* seedlings grown in dark, 0.06, 1.4 or 40 μmol m^−2^ s^−1^ of red light at indicated temperature. Insert: expression of *ATHB2* in WT and *pif4-101pif5-3* grown at 1.4 and 100 μmol m^−2^ s^−1^ of continuous red light at 27 °C. Data normalized to WT expression in dark. Error bars represents s.e.m., *n*=3. (**c**) Hypocotyl measurements of WT grown for 7 days without 1-N-Naphthylphthalamic acid (NPA), with 1 μM NPA or 50 μM NPA at 17 °C or 27 °C at indicated fluence rate of red light. Sample number >28, error bars represents s.e.m. (**d**) Transcript levels of *IAA19* and *IAA29* relative to *IPP2* in 6-day-old WT and *hy5-215* seedlings grown in 1.4 or 40 μmol m^−2^ s^−1^ of continuous red light at indicated temperature. Error bars represents s.e.m., *n*=3.

To test this proposition, we examined the impact of light and temperature on PIF activity by measuring transcript levels of *HFR1* and *XTR7*, two direct targets of PIF4 and PIF5 (ref. 34)^34^. Contrary to our expectations, we observed that at both temperatures, *HFR1* and *XTR7* levels fell in a fluence rate-dependent manner (Fig. 5a), suggesting that these genes do not participate in the temperature-activated switch. In contrast, levels of *IAA19*, *IAA29* and *ATHB2* (direct PIF4 targets) and *SAUR23,* auxin signalling genes previously implicated in PIF4-regulated temperature responses, did correlate with the hypocotyl phenotype; depleting with fluence rate in the cool and rising in the warm (Fig. 5b)^7^,^11^,^20^. Application of the auxin transport inhibitor NPA completely blocked the switch, preventing light-activated hypocotyl elongation at 27 °C, illustrating the prominent role of the auxin pathway in this response (Fig. 5c). Our results are congruent with the recent finding that heat activates PIF4 binding to target promoters by displacing H2A.Z-nucleosomes that would otherwise block PIF4 access^9^. However, we show that the thermal promotion of PIF action is entirely light dependent. Our data therefore validate the model prediction that the photothermal switch is mediated by complete transformation in light control of PIF target genes that are repressed at 17 °C, but activated at 27 °C. Our data also suggest the switch is controlled by a specific gene set that include auxin pathway genes. This view was further strengthened by our finding that HY5 loss led to a de-repression of *IAA19* and *IAA29* mRNA levels, at 17 °C, particularly at higher fluence rates, but had little effect at 27 °C (Fig. 5d). The finding concurs with the recent work showing that HY5 directly opposes PIF action through direct targeting of common genes^26^,^27^.

### Model III predicts continuous light irradiance is required for the switch

Our refined model (Model III) recapitulates experimental data obtained under constant illumination by postulating two fluence rate and temperature-dependent components, X and Y. To further test the model’s predictive power, we considered the case where fluence is delivered in 5- or 15-min pulses that deliver total fluence that matches continuous irradiance controls. The model predicts that under such pulsed light conditions, the switch from inhibition to promotion of hypocotyl extension is not elicited. In experimental pulsed conditions, we observed a modest hypocotyl inhibition response at 17 °C, but these conditions did not generate the switch to elongation at 27 °C (Supplementary Fig. 8c,d). Thus, constant illumination does indeed appear to be required for the temperature-dependent switch. In the model the rate of production of X and Y is directly proportional to the light intensity. This can be achieved in different ways, for example, if the rate of X/Y production is proportional to the flux from Pr to Pfr, or to a photosynthetic output. Our model cannot discriminate between these alternative light input scenarios. However, this analysis confirms the model’s utility as a reliable tool to predict growth under varying light conditions such as those found in the natural environment.

## Discussion

In this study, we used the simple hypocotyl system to interrogate the interaction between light and temperature through the phyB–PIF signalling module. phyB inhibits while PIFs promote hypocotyl extension and control is achieved through reciprocal phyB–PIF cross-regulation. Combining mathematical modelling and experimental approaches allowed us to test current assumptions on light and temperature signalling and generate non-intuitive predictions. We demonstrated that at 17 °C, phyB action was not restricted to the low fluence rate range, but continued to suppress hypocotyl elongation even at relatively high fluence rates (>100 μmol m^−2^ s^−1^). The development of model I exposed a gap in our understanding of how this is achieved. Data combined with model simulation predicted that this regulation could be achieved by ‘X’, a moderator of PIF activity that was fluence rate and possibly temperature dependent (model II). HY5 fulfilled the requirements for X, validating this prediction. A recent study has since shown that HY5 does indeed moderate PIF action by colocalizing to G-box elements of common gene targets^26^,^27^. Interestingly, our data show that HY5 does not appear to control PIF activity at very low fluence rates (for example, 1.4 μmol m^−2^ s^−1^), rather, it becomes increasingly dominant at higher fluence rates when HY5 protein levels increase. HY5 has previously been implicated in cold acclimation and induction of anthocyanin accumulation in response to low temperatures (4 °C)^28^,^29^,^39^. Here we show that HY5 is a more potent suppressor of hypocotyl elongation and PIF-controlled gene expression at the higher temperature of 17 °C. However, this control is markedly reduced at 27 °C.

Unexpectedly, we found that the ‘classical’ hypocotyl fluence rate response is completely temperature dependent. At cooler temperatures, red light strongly inhibits hypocotyl extension, but at 27 °C, over a minimal fluence rate, light promotes elongation. We showed that the warm temperature behaviour does not simply arise from alterations in phyB or PIF levels. Rather this effect results from a light-dependent alteration in PIF activity. Interestingly, this light-mediated control is executed through a specific gene subset that includes auxin response genes. Our data therefore offer a plausible solution to the long standing, but unexplained observation that light is required for auxin promotion of hypocotyl elongation at high temperatures^6^,^8^. Until now, this finding appeared to be at odds with the well-established repressive role of light in this response.

To gain an understanding of how light and temperature convergence changes the signalling characteristics of the phyB–PIF module, we developed a photothermal model (Model III). This model, which incorporated temperature-dependent parameters, was able to match the physiological data by including the temperature-activated components X and Y. However, Model III could only simulate the temperature-induced switch from hypocotyl inhibition to elongation when these variables were also fluence rate dependent, suggesting a requirement for continuous irradiation. Indeed, when simulating pulsed light conditions, the model could no longer match the data. We tested this prediction by supplying seedlings with the same total fluence of red light as either continuous irradiation or 5- or 15-min discrete pulses. While we saw a minor impact of the pulse treatment at 17 °C, we could not generate the switch to promotion at 27 °C. This experiment verified our model and suggested that at both temperatures, but particularly at 27 °C, continuous light is required to generate the hypocotyl response. High fluence rate control could be mediated either through a biochemical mechanism acting as a Pr to Pfr flux counter or else via a phyB-independent mechanism such as photosynthesis. Sucrose-induced hypocotyl elongation has recently been shown to be dependent on PIFs, indicating that increased photosynthesis is a plausible candidate mechanism^40^,^41^. The models can achieve the requisite fluence rate-dependent regulation in a phyB dependent or a phyB-independent manner and so does not discriminate between these two possibilities.

Our finding that light activates PIF-dependent gene expression at 27 °C aligns with the recent report that PIF4 access to the *FT* promoter is facilitated by temperature-induced chromatin modification^31^. This is achieved through heat-induced expulsion of the H2A.Z histone variant from nucleosomes that, when present, blocks transcription. Other studies have implicated histone acetylation in light-activated gene expression^42^,^43^,^44^. Indeed, PIF3 was recently shown to repress photosynthetic gene transcription through histone deacetylase 15 (ref. 45)^45^. It is therefore possible that the dual effect of light and temperature on transcriptional activation is mediated through dynamic changes in chromatin structure and access to DNA. Alternatively, the boost in transcription could be achieved by one or more transcription factors that work cooperatively with PIFs. A recent study demonstrated that BZR1 heterodimerizes with PIF4 and is required for heat-induced hypocotyl extension^5^. It is, however, uncertain that BZR1 would fulfil the requirements for ‘Y’ as light does not appear to promote BZR1 action, rather, it leads to the accumulation of the inactive, phosphorylated form of BZR1 (ref. 46)^46^. Nonetheless, the observed elevated expression of gene subset could be achieved through an analogous co-operative heterodimerization mechanism.

In the natural environment, plants have to react simultaneously to changing light and temperature cues. This study illustrates how signal integration can culminate in unforeseen molecular signalling outputs. We have shown that temperature does not affect all reactions equally; rather, different parameters have specific temperature sensitivities, which is important to deliver distinct outcomes. Our model-based approach has shown that light imposes strict control on PIFs, by enlisting regulators of PIF activity. In cool conditions, light promotion of HY5 levels is particularly important to suppress PIF action at higher fluence rates. As temperature rises, light promotes PIF activity resulting in fluence rate-dependent promotion of hypocotyl elongation. This non-intuitive switch in light-induced behaviour was predicted by mathematical models and validated experimentally.

## Methods

### Growth conditions

For all experiments, seeds were surface sterilized and sown on agar plates containing ½ Murashige and Skoog media. After 3–4 days stratification at 4 °C, the seeds were exposed to 2 h of white light at 20 °C followed by 22h incubation in darkness before moved in to the experimental conditions.

### Plant material

The *phyB-9*, *phyB-401*, *hy5-215*, *hfr1-101*, *pif3-3*, *pif4-101*, *pif4-2, pif5-3* double and multiple mutants as well as *35S:PIF4-HA* and *35S:PIF5-HA* are all in the Columbia background and have been described previously^22^,^47^,^48^,^49^,^50^,^51^,^52^. *35S:HY5-HA* is in the Wassilewskija background^53^ and *della4* (*rgl1-1;rgl2-1;gai-6;rga-2*) in Landsberg^54^. *35S:PIF3-LUC* has been described previously^55^ and for creation of *35S:PIF4-LUC*, *PIF4* coding sequence was inserted to the *35S:PIF3-LUC* pPCV812 vector as an *Xba*I–*Sma*I DNA fragment, replacing *PIF3*-coding sequence. *PHYB:PHYB-LUC* was created by replacing the *Nicotiana PHYB* counter parts, cloning the *Arabidopsis PHYB* promoter as *Hin*dIII–*Bam*HI and *PHYB* cloning region as *Sma*I–*Stu*I DNA fragments into the *NtPHYB:PHYB-LUC* pPCV812 binary vector^56^.

### Immunoblotting and quantitative PCR

For immunoblotting, total protein samples from seedlings grown as indicated in the main text were separated on a 10% SDS–polyacrylamide electrophoresis gel and transferred to a nitrocellulose membrane as previously described^6^. For protein quantification, three biologically independent experiments were analysed using anti-HA-HRP (3F10, Roche) and anti-UGPase (AGRISERA) at a dilution of 1:1,000. ImageJ software was used for PIF4-HA quantification, which was normalized to the dark sample at 17 °C. An uncropped scan corresponding to Fig. 2a is shown in Supplementary Fig. 9. For HY5-HA quantification, Metamorph (Molecular Devices) was used and normalized to a reference sample.

Quantitative PCR was performed as previously described^6^. In short, the samples were harvested into RNAlater (Sigma), and total RNA was extracted using RNeasy Plant Mini Kit (Qiagen). cDNA synthesis was performed using SuperScript VILO cDNA Synthesis Kit (Invitrogen). Primers used for qPCR are listed in Supplementary Table 2.

### Protein quantification using Luciferase assay

For protein quantification using Luciferase, ~100 mg of 6-day-old seedlings (*PHYB:PHYB-LUC, 35S:PIF4-LUC* and *35S:PIF3-LUC)* were harvested into liquid nitrogen. Samples were ground in liquid nitrogen and proteins were extracted in 200 μl Promega Cell Culture Lysis Reagent complemented with 50 μM of MG132, MG115, ALLN, PS1 and 1 × Complete protease inhibitor cocktail, EDTA-free (Roche). The samples were centrifuged at 14,000 r.p.m. for 10 min at 4 °C to remove debris. In total, 50 μl of each sample was then added to a 96-well plate and analysed using MicroLumatPlus LB96V Microplate Luminometer with the Luciferin-based Reporter Lysis Buffer (Promega). To determine the total protein content of the samples, 50 μl of each sample was first washed using the Compat-Able Protein Assay Preparation Reagent Set (Thermo) to remove the detergent according to the manufacturer’s instructions. Total protein was then assayed using Pierce BCA kit (Thermo) according to the manufacturer’s instructions and absorbance was measured at 562 nm. A dilution series using bovine serum albumin was used as a reference to determine the sample protein content.

## Author contributions

K.J.H., H.J., discovered the photothermal switch. K.J.H., H.J., H.J.J. designed the research. H.J., J.F., J.R.H., K.S. performed the experiments. K.J.H., R.G. and H.J.J. built and analysed the mathematical models. K.J.H., H.J., H.J.J., R.G. analysed the data and wrote the manuscript. K.J.H., R.G. provided overall guidance on the project.

## Additional information

**How to cite this article:** Johansson, H. *et al.* *Arabidopsis* cell expansion is controlled by a photothermal switch. *Nat. Commun.* 5:4848 doi: 10.1038/ncomms5848 (2014).

## Supplementary Material

Supplementary Information Supplementary Figures 1-9, Supplementary Tables 1-2, Supplementary Methods and Supplementary References
