# Supplementary material for: Arabidopsis cell expansion is controlled by a photothermal switch
Source: Nat Commun. 2014 Sep 26;5:4848. doi: 10.1038/ncomms5848 (PMC4200516; doi:10.1038/ncomms5848)
Supplement: Supplementary Information — Supplementary Figures 1-9, Supplementary Tables 1-2, Supplementary Methods and Supplementary References [file ncomms5848-s1.pdf]

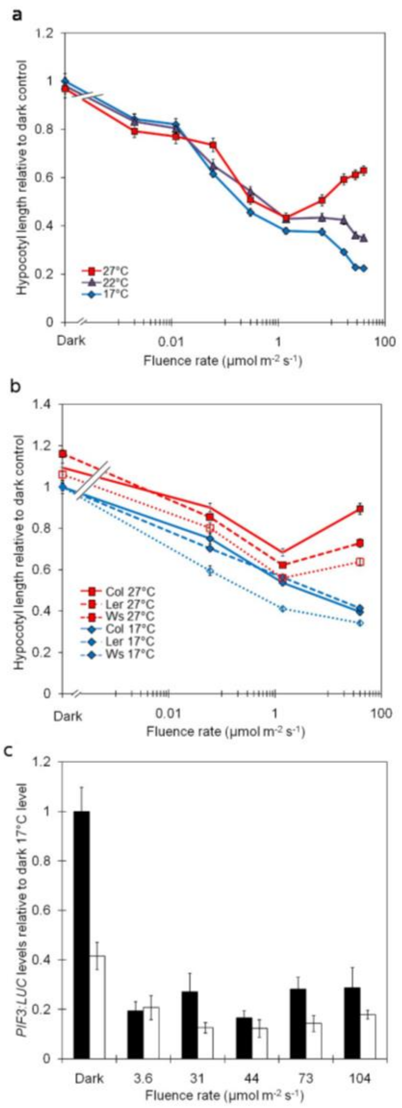

Supplementary Figure 1: Hypocotyl fluence response curves and quantification of PIF protein and transcript levels

a, Fluence rate response curves measuring hypocotyl elongation of 7 days old WT seedlings grown in continuous red light at 17°C, 22°C and 27°C . Sample number >16, error bars represent s.e.m. **b,** Fluence rate response curves measuring hypocotyl elongation of 7 day old seedlings grown in continuous red light of Col, Ler and Ws at 17°C and 27°C. Sample number >19, error bars represent s.e.m. **c,** Quantification of PIF3-LUC grown at 17°C (black) and 27°C (white) at indicated fluence rates. Error bars represent s.e.m., n=3.

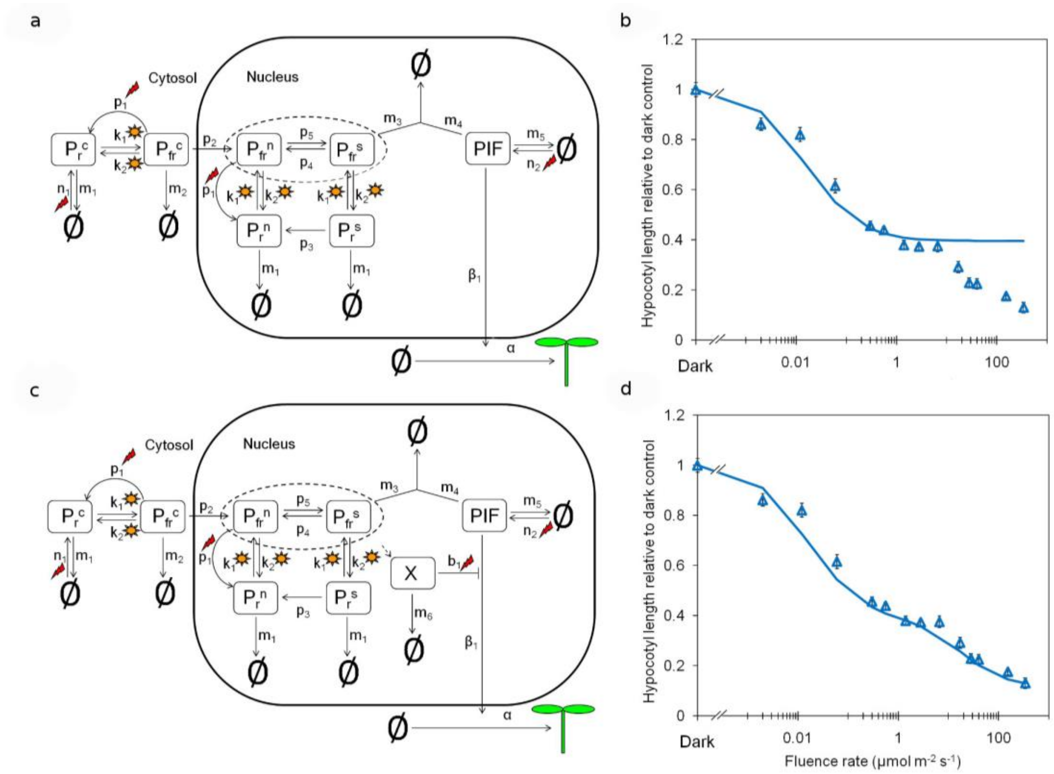

Supplementary Figure 2: Model I and Model II

a, Schematic of Model I including formation and transportation of phyB from the cytosol to the nucleus into the nuclear speckles. The dashed line indicates that both nuclear and speckle pools of Pfr participate in the mutual degradation with PIF. Hypocotyl elongation is regulated by activation of PIF. **b,** Experimental data at 17°C (blue diamond) compared with simulation of Model I (solid line). **c,** Schematic of Model II including a new X component which inhibits PIF activity in a fluence rate dependent manner. X is coupled to light intensity via a phyB-independent process such as photosynthesis or via a phyB-dependent process (see text for details). **d,** Experimental data at 17°C (blue diamond) compared with simulation of Model II (solid line). Error bars represent s.e.m., n > 17.

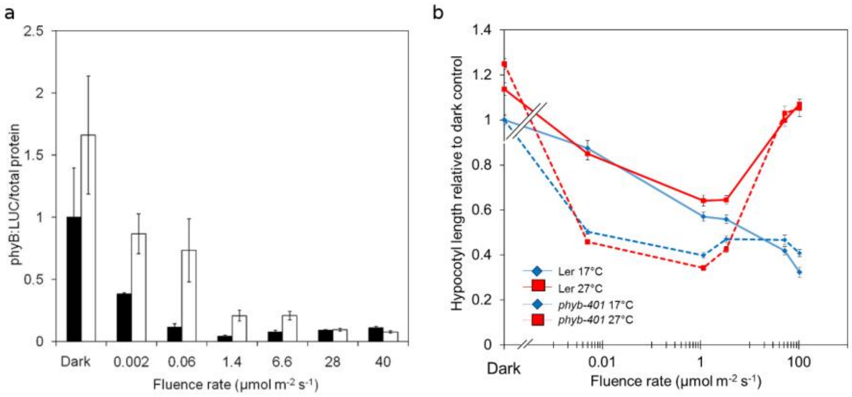

Supplementary Figure 3: PhyB protein levels and phenotype of *phyB-401* over a range of fluence rates.

a, Quantification of phyB-LUC grown at 17°C (black) and 27°C (white) at indicated fluence rates of light. Error bars represent s.e.m., n=3. **b,** Fluence rate response curves measuring hypocotyl elongation of 7 day old seedlings under red light of WT and *phyB-401* at 17°C and 27°C . Error bars represent s.e.m., n > 24.

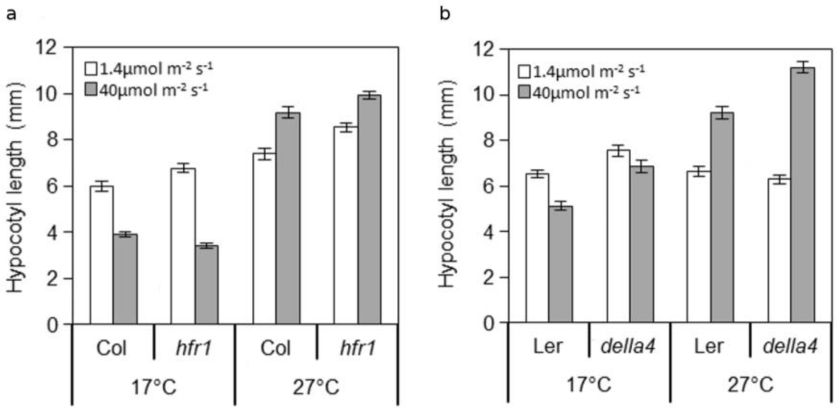

Supplementary Figure 4: Analysis of *hfr1* and *della4* mutants

a, Hypocotyl measurements of 7 day old WT and *hfr1-101* seedlings grown at indicated temperature and fluence rate of continous red light. Sample number > 16, error bars represents s.e.m. **b,** Hypocotyl measurements of WT and the *della4* mutant grown as in **a**. Sample number > 21, error bars represents s.e.m.

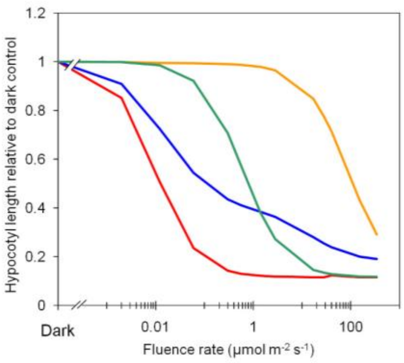

Supplementary Figure 5: Importance of reciprocal phy-PIF regulation in Model II

Simulation of Model II (blue line) compared to Model II without PIF-dependent Pfr degradation (m_3=0) (red line), Model II without Pfr-dependent PIF degradation (m_4=0) (orange line) and Model II without any Pfr or PIF dependent degradation (green line).

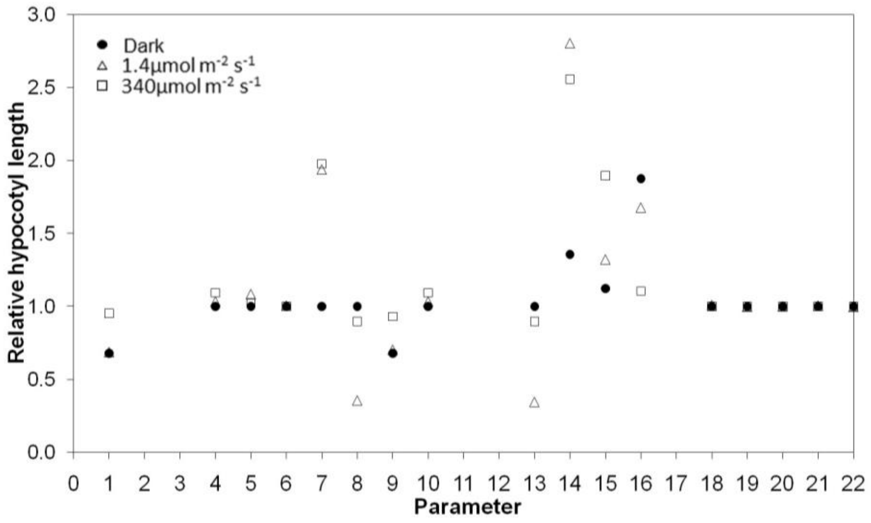

Supplementary Figure 6: Temperature Sensitivity Analysis

Graphical representation of the local temperature sensitivity analysis for each parameter within the Model II (See Supplementary Table 1 for parameter identification). This shows the effect of $Q_{10} = 2$ for each parameter individually in the dark, at 1.4µmol m^{-2} s^{-1} and 340µmol m^{-2} s^{-1}.

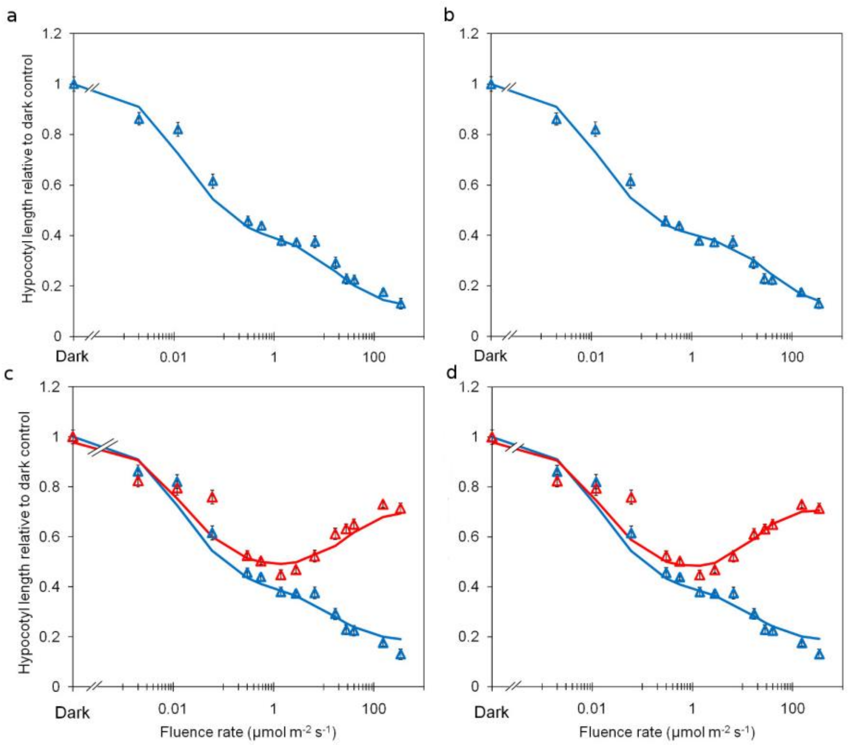

Supplementary Figure 7: X and Y dependence on phyB in Model II and Model III

Experimental data at 17°C (blue triangle) compared with simulation of Model II at 17°C (blue line) when **a,** concentration of X is phyB-dependent. **b,** concentration of X is PhyB-independent. Experimental data at 17°C (blue triangle) and 27°C (red triangle) compared with simulation of Model III at 17°C (blue line) and 27°C (red line) when **c,** concentration of Y is phyB-dependent. **d,** concentration of Y is PhyB-independent. Error bars represents s.e.m., n > 17.

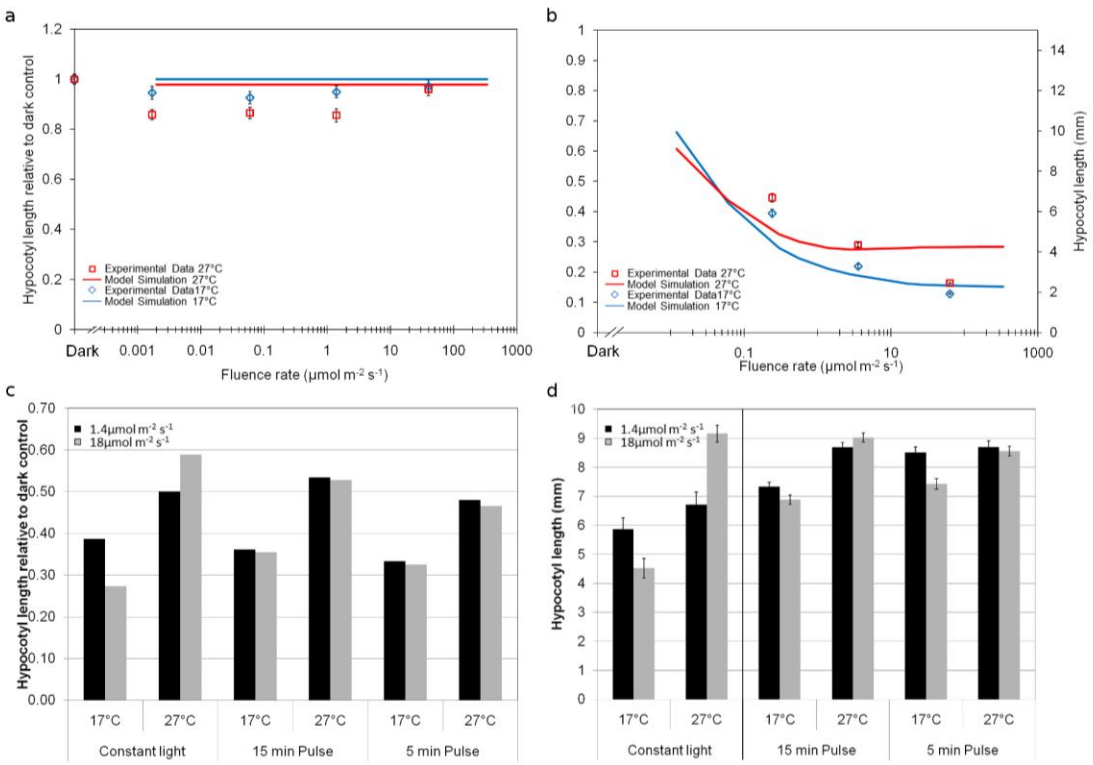

Supplementary Figure 8: Comparison of experimental data and Model III simulations.

Comparison of 7 days old *phyB-9* **a**, and *pif4pif5* **b**, seedlings grown in continuous red light at 17°C and 27°C with simulated mutant data from Model III at 17°C and 27°C. Sample number > 19 (a) and > 16 (b). Error bars represents s.e.m. Simulated hypocotyl length from Model III **c**, and hypocotyl measurements **d,** of seedlings grown in constant red 1ight at 1.4µmol m^{-2} s^{-1} and 18µmol m^{-2} s^{-1} or given equivalent amount of total light provided by 5 or 15 minute hourly pulses. Error bars represents s.e.m., n > 18.

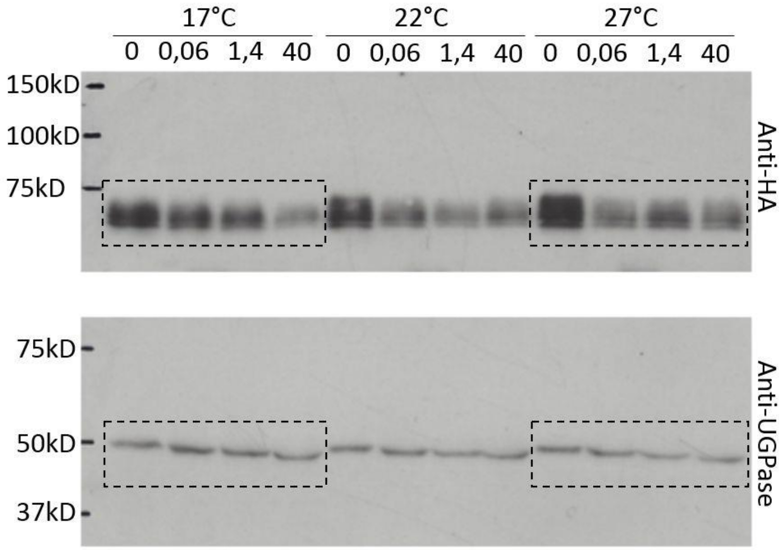

Supplementary Figure 9: Scan of PIF4-HA and UGPase western blot.

Boxes indicate the areas cropped and displayed in Figure 2a.

Ref.	Parameter	Description	Estimated Parameter Value (per min)	Q_{10}
1	g_1	M-M constant of PIF activity (X pathway)	0.9520	1.6
2	g_2	M-M constant of PIF activity (Y pathway)	4.1410	1.0
3	g_3	M-M constant of Y pathway	1.2734	1.0
4	b_1	Inhibition constant for X	0.2673	3.1
5	m_1	Degradation of Pr	0.0006	1.0
6	m_2	Degradation of P_{fr}^c	0.0024	1.0
7	m_3	PIF-dependent deg of Pfr	0.0171	1.4
8	m_4	Pfr-dependent deg of PIF	6.8380	1.3
9	m_5	Degradation of PIF	0.5830	1.5
10	m_6	Degradation of X	0.9860	1.0
11	m_7	Degradation of Y	0.4060	1.0
12	m_8	Growth inhibition constant	1.0000	1.0
13	n_1	P_r^c formation	0.0009	1.9
14	n_2	PIF Formation	0.4370	1.9
15	α	Basal rate for growth	0.1200	1.5
16	β_1	PIF reaction rate (X pathway)	2.0800	1.0
17	β_2	PIF reaction rate (Y pathway)	1.9400	5.0
18	p_1	Dark reversion	0.1000	2.0
19	p_2	Translocation P_{fr}^c to P_{fr}^n	1.0000	1.0
20	p_3	Translocation P_r^s to P_r^n	1.0000	1.0
21	p_4	Translocation P_{fr}^s to P_{fr}^n	1.5100	1.0
22	p_5	Translocation P_{fr}^n to P_{fr}^s	3.9800	1.0

Supplementary Table 1: Parameter set for Model III

Table 1 gives a brief description of each parameter used in Model III and the optimized value used in simulation of all models. The Q_{10} value indicates the temperature dependence of each parameter. A parameter identifiability analysis (by means of a Gaussian approximation of the posterior probability with a flat prior) shows that only 3 of the 22 parameters (namely g_1, m_5 and p_3) are non-identifiable.

Primer	Sequence (5’-3’)
XTR7-F	ACACATCATATTCTTGGTGGAC
XTR7-R	TAGATCCTCATGGGTTGACTC
SAUR23-F	CTCTCATACTTGAACCAGCCT
SAUR23-R	CATTGATGAAAGTATCTTCGGGAC
PIF5-F	ACCATGGGTAACAAATCGAG
PIF5-R	GAAGCTTTATCTGTTCTGCTG
PIF4-F	GTATCTCTATCAGATGCAATCGG
PIF4-R	CTTGCAAAGCCTTCATTCTC
IAA19-F	TGGCCTTGAAAGATGGTGAC
IAA19-R	TTGCATGACTCTAGAAACATCCC
IAA29-F	GATGTTACATGGAAGATCTTTGCG
IAA29-R	CCGATTTGAACGCCTATCCT
HFR1-F	AGAGAAGTTCCTTCAGTTACTCG
HFR1-R	AACCTTGTCCGTCTTGTGAC
IPP2-F	GTATGAGTTGCTTCTCCAGCAAAG
IPP2-R	GAGGATGGCTGCAACAAGTGT
ATHB2-F	GAGGTAGACTGCGAGTTCTTAC
ATHB2-R	TTCTCTTCCGTTAGATTCTCGCA

Supplementary Table 2: Primers used for transcriptional analysis

Supplementary Methods:

1. Introduction

It is well established that plants grown in the dark have longer hypocotyls than those grown in the light. As fluence rate increases the extent of hypocotyl elongation decreases[1]. It has also been shown that this effect is temperature dependent such that increasing temperature leads to greater

hypocotyl growth. Our new data in the model plant *Arabidopsis thaliana* illustrates that light control of hypocotyl elongation exhibits distinct characteristics that alter with temperature. At moderate ambient temperatures, e.g. 17°C, we observe the classical fluence rate dependent inhibition of hypocotyl extension. But at 27°C, fluence rates above 1.4 μmol m^{-2} s^{-1} lead to incremental increases in hypocotyl elongation (Fig. 1, main text). The aim of our model was to determine whether a biochemical circuit could be established which mimics this hypocotyl behaviour through the interaction of temperature and light signalling. This would allow us to take a systems level approach to understand how temperature can generate the comprehensive switch in light control of cell expansion. To do this we took as our starting point a published ordinary differential equation (ODE) phyB model and extended this to incorporate hypocotyl regulation through PHYTOCHROME INTERACTING FACTORS (PIFs). This mathematical model was then further refined using qualitative and quantitative experimental data. In our initial model (Model I) we held the temperature fixed at 17°C, which allowed us to model accurately the light input into the system. Subsequently we developed a second model (Model II) that included the effects of temperature and then a final Model III that was able to fully recapitulate the temperature switch.

1.1 Details of Model I

PhyB is formed in an inactive state within the cytosol (Pr). Following absorption of red light Pr is photo-transformed into its active state, Pfr (maximal conversion occurs at 660nm). Pfr can be photoconverted back into Pr upon absorption of far-red light, with maximal conversion at 730nm[2], and also converts back to Pr in the dark, a process referred to as dark reversion (dr). The phyB dynamic model proposed by Rausenberger *et al.* (2010)[3] considers the concentrations of

six separate intracellular pools of phyB, namely diffusely distributed inactive (Pr) and active (Pfr) conformers of phyB localized within the cytosol, nucleus and nuclear speckles (P_r^c, P_{fr}^c, P_r^n, P_{fr}^n and P_r^s, P_{fr}^s, respectively). As hypocotyl elongation is controlled by a dynamic balance between PIF and phyB, we extended the Rausenberger *et al.* model by incorporating the mutual interaction between Pfr and PIF and adding a direct output of hypocotyl length. We refer to this as Model I and it is illustrated in Supplementary Figure 2a. The physical interaction of active phyB and PIF induces proteolytic degradation of both phyB and PIF, creating a mutually-negative feedback loop[4-6]. It has yet to be established whether the Pfr:PIF interaction occurs at all points within the nucleus or only within the nuclear speckles[3]. In Model I we therefore make the assumption that all Pfr within the nucleus (P_{fr}^n and P_{fr}^s) can react with PIF. In addition Model I includes synthesis and degradation of PIF. We use Michaelis-Menten kinetics to describe the positive correlation between PIF concentrations and hypocotyl length.

The transport of phyB within the cell is assumed to be very fast with respect to other dynamics; therefore we can model phytochrome interactions using a non-spatial ODE model. Based on previous work[3] and these observations we can construct the model as follows:

$$\frac{dP_r^{\,c}}{dt} = n_1 - (m_1 + k_1)P_r^{\,c} + (p_1 + k_2)P_{fr}^{\,c}, \tag{1}$$

$$\frac{dP_{fr}^{\,c}}{dt} = k_1 P_r^{\,c} - (k_2 + p_1 + m_2 + p_2)P_{fr}^{\,c}, \tag{2}$$

$$\frac{dP_r^{\,n}}{dt} = (k_2 + p_1)P_{fr}^{\,n} + p_3 P_r^{\,s} - (k_1 + m_1)P_r^{\,n}, \tag{3}$$

$$\frac{dP_{fr}^{\,n}}{dt} = p_2 P_{fr}^{\,c} + k_1 P_r^{\,n} + p_4 P_{fr}^{\,s} - (k_2 + p_1 + p_5)P_{fr}^{\,n} - m_3 P_{fr}^{\,n} PIF, \tag{4}$$

$$\frac{dP_r{}^s}{dt} = k_2 P_{fr}{}^s - (k_1 + p_3 + m_1) P_r{}^s, \tag{5}$$

$$\frac{dP_{fr}{}^s}{dt} = k_1 P_r{}^s + p_5 P_{fr}{}^n - (k_2 + p_4) P_{fr}{}^s - m_3 P_{fr}{}^s PIF, \tag{6}$$

$$\frac{dPIF}{dt} = n_2 - m_4 PIF\left(P_{fr}{}^n + P_{fr}{}^s\right) - m_5 PIF, \tag{7}$$

$$HL = \alpha + \beta_1 \frac{PIF}{g_1 + PIF}, \tag{8}$$

where parameters n_i, m_i represent the rate constants of synthesis and degradation respectively; k_i are phyB photoconversion rates; p_1 is the dark reversion rate and the remaining p_i are constants of translocation between the cytoplasm, nucleus and nuclear speckles; g_i are Michaelis-Menten constants. β_i is the maximum rate achieved by PIF activity upon the growth rate. α is the basal hypocotyl length (i.e. when there is no PIF present). Dark reversion does not affect the levels of $P_{fr}{}^s$ as it has been suggested that Pfr is stabilized within the nuclear speckles, protecting it from dark reversion[3]. Pfr rapidly induces the phosphorylation of PIF (triggering degradation) however PIF induces the degradation of Pfr through a different mechanism, allowing for the differing degradation kinetics of PIF and phyB (m_3 and m_4) following their interaction (Pfr:PIF)[4-6].

1.2 Parameter Optimization

All experimental data were collected from seedlings grown in constant red light (660nm) conditions for 7 days, therefore the concentrations of Pr, Pfr and PIF were at equilibrium for each light intensity recorded. This supports our use of a steady-state model; hence the ODEs were

solved explicitly using parameter values constrained to available hypocotyl and protein data. All experimental data have been normalized to the dark values, i.e. dark grown hypocotyls have length 1. The parameter values were estimated within valid biological ranges by various methods: k_1 and k_2 are the rate of photoconversion between Pr and Pfr forms of phyB in red and far-red light respectively. These parameters were calculated explicitly by $k_1=N_\lambda\sigma_r$ and $k_2=N_\lambda\sigma_{fr}$ where N_λ denotes the photon flux at wavelength λ (660nm) and $\sigma_{r/fr}$ represents the photoconversion cross-sections of red/far-red pigment [7]. Parameters m_1 (P_r^c degradation), m_2 (P_{fr}^c degradation), p_1 (rate of dark reversion), p_2 (translocation from P_{fr}^c to P_{fr}^n), p_3 (translocation from P_r^s to P_r^n), p_4 (translocation from P_{fr}^s to P_{fr}^n) and p_5 (translocation from P_{fr}^n to P_{fr}^s) are all within the range demarcated by the Rausenberger *et al.* model [3]. Values for the remaining parameters n_1 (P_r^c formation), n_2 (PIF formation), m_3 (PIF-dependent degradation of Pfr), m_4 (Pfr-dependent degradation of PIF), m_5 (PIF degradation), β_1 (PIF activation rate), g_1 (Michaelis-Menten constant) and α (basal growth rate) were optimized through simulated annealing least-square fitting to the hypocotyl data and the underlying PIF and phyB levels of Columbia wild type (Col WT) at 17°C (Fig. 1a and Supplementary Fig. 1c, 3a). Supplementary Figure 2b illustrates the output of Model I with these optimized parameters. This set of parameters was then used in further analysis of the model and is listed in Supplementary Table 1. All simulations were performed using the MATLAB software from MathWorks, Inc. and MAPLE 15 software from Maplesoft, Inc.

1.3 Details of Model II

We observed that at 17°C Model I provided a good fit to the wild type experimental data up to 1.4 µmol m^{-2} s^{-1}; as illustrated by Supplementary Figure 2b. Above this fluence rate, Model I predicts a plateau in hypocotyl growth; this is in accordance with the Rausenberger *et al.* model[3]. However our experimental data (Fig. 1a) illustrate that further hypocotyl inhibition occurs above the 1.4 µmol m^{-2} s^{-1} threshold. In Model I the fluence response of hypocotyl growth is directly linked to PIF levels (Eq. 8); which, in turn, are dependent on the levels of active phyB (Eq. 7). Therefore one possibility was that increased hypocotyl inhibition could be achieved through increased phyB levels promoting further PIF degradation. However, our experimental data clearly indicate the decrease in total phyB levels stabilizes to low levels of phyB at around 1.4 µmol m^{-2} s^{-1}, regardless of further fluence rate increases (Supplementary Fig. 3a). This plateauing of phyB levels implies there could be no further reduction of PIF levels after 1.4 µmol m^{-2} s^{-1}. This hypothesis was supported by western blots of the PIF3 protein, which is acutely sensitive to phyB Pfr levels [8]. Like phyB, PIF3 depletes to a low stable level that is maintained at high fluence rates (Supplementary Fig. 1c). However, PIF4-HA and PIF4-LUC show a continued depletion as fluence rate increases (Fig. 2a, b). This implies that PIF quantity does not correlate with the hypocotyl response, which in turn indicates that Model I is incapable of achieving further hypocotyl inhibition at high fluence rates. To test this we ran the model through 200,000 parameters sets selected using Latin Hypercube Sampling (LHS). None of these parameter sets matched the data, suggesting that an additional component(s) was required to deliver the requisite behaviour at high fluence rates.

As phyB and PIF levels did not correlate with hypocotyl length at higher fluence rates, we surmised that altered PIF activity could be an important contributory factor. Reinforcing this

proposition, PIFs are known to be regulated post-translationally[9-11]. In accordance Model I was extended to incorporate component X, which elicits fluence rate-dependent inhibition of PIF activity, to create Model II (Supplementary Fig. 2c).

The fluence rate component of X regulation can be achieved either through phyB dependent (Eq. 10) or independent (Eq. 11) processes. In both cases the rate of production of X is made directly proportional to the light intensity. For a phyB dependent process, this is achieved by making the production rate proportional to Pr, i.e., to the flux from Pr to Pfr. A simple biochemical mechanism by which such a flux counter can be implemented would involve the creation of a molecule of X each time that a molecule of Pr changes to Pfr; this is explicitly modelled by the first term on the right hand side of Eq. 10. For a phyB independent mechanism, X is assumed to depend on a factor involved, for example, in photosynthesis; in the absence of information about which factor this is and of the specific pathway/s involved, this case can only be modelled by making the production rate of X directly proportional to the light intensity (see first term on the right hand side of Eq. 11). In both cases a light-independent decay of X is introduced such that X is in steady-state. Note that assuming the production rate of X to be directly proportional to Pfr does not lead to the same case as above since Pfr levels are not directly proportional to the light intensity but rather saturate at low light intensities. The steady-state hypocotyl length for Model II and the two possible type of kinetic equations for X can be described by

$$HL = \alpha + \beta_1 \frac{PIF}{g_1 + PIF} \frac{b_1}{b_1 + X}, \tag{9}$$

$$\frac{dX}{dt} = k_1 (P_r^{\,n} + P_r^{\,s}) - m_6 X, \tag{10}$$

$$\frac{dX}{dt} = N_\lambda - m_6 X. \tag{11}$$

In order to distinguish between the two possible model inputs for X we initially estimated b_1 (inhibition constant of X) and m_6 (X degradation) using simultaneous simulated annealing least-square fitting to hypocotyl growth data and the underlying PIF and phyB levels of Columbia wild type (Col WT) at 17°C (Fig. 1a and Supplementary Fig. 1c, 3a). Both equations for the concentration of X have the same number of unknown parameters and as they are being compared to the same data the standard method for model selection, Bayesian Information Criterion (BIC), can be reduced to χ^2 values. The χ^2 values are 0.020 and 0.018 for phyB-dependent and independent X respectively (Supplementary Fig. 7a, b), showing that we cannot differentiate between these two possible methods for regulating X. Purely as a point of reference, hereafter X was modelled as phyB-dependent.

The modified model including X (Model II) is illustrated in Supplementary Figure 2c, d. Comparing Supplementary Figure 2b and 2d we show that X affects hypocotyl elongation at high fluence rates, through suppression of PIF activity. Most importantly inclusion of X leads to a model which agrees with the experimental data over the whole range of fluence rates tested.

1.4 Including Temperature Effects in Model II

Our next aim was to integrate the temperature and light signals affecting hypocotyl elongation into one model, which necessitated the extension of Model II to describe the fluence dependent hypocotyl elongation seen at 27°C. A possible hypothesis was that this switch from inhibition to promotion of hypocotyl elongation was primarily due to dark reversion (dr); the light-

independent conformational change from Pfr to Pr. Earlier model analysis showed that the light driven photochemical reactions that establish Pr:Pfr ratio fail to deliver the characteristic fluence response curve over a large fluence range[3]. Inclusion of dr corrected this deficiency, highlighting the importance of dr in light intensity sensing. Dark reversion is known to be temperature dependent[12]; hence it is possible that changes in dr kinetics could drive the thermal switch in the fluence rate response. Countering this suggestion, dr is only thought to be influential at the low fluence rate range[13], while the temperature induced changes are observed at higher fluence rates. Nonetheless we tested whether dark reversion was required for the switch to address this uncertainty. Here we examined the *phyB-401* mutant that carries a G-E amino acid substitution at position 564. This substitution increases the spectral range of activity and blocks dr[14]. Concurring with previous results, the *phyB-401* mutation led to extreme hypersensitivity to red light, evidenced by the exaggerated response to very low fluence rate light (Supplementary Fig. 3b). This was observed at both 17°C and 27°C. *phyB-401* exhibited a clear switch response, as at 27°C hypocotyl elongation was driven by fluence rate. These results eliminate dr as a principal regulatory factor in the photothermal switch.

All reactions are affected by temperature to some extent, we therefore postulated that the inclusion of temperature sensitivity to multiple parameters within Model II could illicit the required switch in hypocotyl behaviour. Hence we assigned each parameter a Q_{10} value. A Q_{10} value measures the change in a rate constant due to an increase from an initial temperature, T_1, to the final temperature, T_2:

$$Q_{10} = \left(\frac{k_{T_2}}{k_{T_1}}\right)^{\frac{10}{T_2 - T_1}}. \qquad \textbf{(12)}$$

In all of our models the temperature increase was 10°C. Hence a Q_{10} value of 1 implies no temperature sensitivity in the rate of reaction whereas a Q_{10} value of 5 infers a fivefold increase in reaction rate over a 10°C temperature increase[15,16]. Biologically relevant Q_{10} values are thought to be in the range of 1-5[15], the most commonly found is Q_{10} = 2 which is the familiar doubling of the rate constant for every 10°C increase in the temperature[17]. Using this measure of temperature sensitivity we aimed to identify the reactions where a change in temperature dependence has a noticeable effect on the overall hypocotyl growth.

In darkness, when it is entirely in the Pr form and found only in the cytoplasm, phyB levels are slightly elevated at 27°C compared with 17°C, indicating either n_1 (formation) or m_1 (degradation) of Pr is temperature dependent. A moderate Q_{10} value has therefore been assigned to Pr formation, reproducing the temperature dependent increase in phyB. Similarly PIF4 expression and protein levels have been shown to rise with temperature at all fluence rates and accordingly we have designated n_2 (PIF formation) and m_5 (PIF degradation) as potentially temperature dependent (Fig. 2a, b)[18-20]. Our data show m_3 (PIF- dependent degradation of Pfr) and m_4 (Pfr- dependent degradation of PIF) are also temperature sensitive. The gradient of reduction in phyB levels, a PIF-dependent process, is greater at 27°C (-1.04 $\mu mol^{-1}\ m^2\ s^1$) than at 17°C (-0.68 $\mu mol^{-1}\ m^2\ s^1$) (Supplementary Fig. 3a) indicating the PIF-dependent degradation of phyB (m_3) is effected by temperature. Likewise, fluence rate (and phyB Pfr-dependent) depletion of PIF protein (m_4) is temperature sensitive, with gradients of -0.038 $\mu mol^{-1}\ m^2\ s^1$ at 27°C and -0.014 $\mu mol^{-1}\ m^2\ s^1$ at 17°C (Fig. 2a, Supplementary Fig. 1c). In each case the gradients were measured between the dark value and the fluence rate at which phyB/PIF levels plateau. As dark reversion, p_1, known to be temperature dependent we assigned it a Q_{10} value of 2; however due to

dark reversion only affecting the hypocotyl length at very low light intensities, we anticipate that it will have little impact at the higher fluence rates (Supplementary Fig. 3b)[12,21]. The basal rate of cell expansion, α, in the model is also reported to potentially be temperature dependent[17] and we will therefore assign it a Q_{10} value. Finally our genetic data suggested that the activity of HY5 (X in Model II) may decrease with temperature; however HY5 protein levels do not. This would indicate that b_1 (the inhibition constant of X) is temperature dependent and therefore we included this as a possible temperature dependent parameter.

To identify other potential temperature parameters we performed a local sensitivity analysis on the effects of temperature within the system. For this analysis we recorded the extent of hypocotyl elongation at the three crucial fluence rates (dark, 1.4 μmol m^{-2} s^{-1} and 340 μmol m^{-2} s^{-1}) in the absence of temperature dependence. Each separate parameter was doubled (Q_{10}=2) and the simulated hypocotyl length was then recorded and normalized against hypocotyl length at 17°C. This analysis was used to identify those parameters where increasing temperature sensitivity generates a light dependent change in hypocotyl length (Supplementary Fig. 6). As anticipated, in darkness, only parameters involved in determining PIF levels and activity, n_2 (PIF formation), m_5 (PIF degradation), β_1 (PIF activation rate) and α (basal growth rate), caused a change in hypocotyl length. However we do not observe significant temperature dependence in dark grown hypocotyls in the data (Fig. 1a).

Local temperature sensitivity analysis identified a small set of potentially interesting parameters which display a large light dependent temperature effect on hypocotyl length. At low fluence rates (1.4 μmol m^{-2} s^{-1}) m_4 (Pfr- dependent degradation of PIF), n_1 (P_r^c formation), n_2 (PIF

formation) and g_1 (Michaelis-Menten constant) have a significant effect. However in the data we observe a moderate reaction to temperature increase at this fluence rate, so this was taken into account when assigning Q_{10} values to these parameters. At high fluence rates n_2 (PIF formation), m_3 (PIF- dependent degradation of Pfr) and α (basal growth rate) were identified as temperature sensitive parameters. Unexpectedly all other parameters were relatively insensitive to changes in temperature dependence. This sensitivity analysis supports the addition of temperature dependence to n_1 (formation rate of P_r^c), n_2 (formation rate of PIF), p_1 (dark reversion rate), m_3 (PIF- dependent degradation of Pfr) and m_4 (Pfr- dependent degradation of PIF), m_5 (PIF degradation) and α (basal growth rate), parameters previously identified to potentially be temperature dependent. In addition the temperature sensitivity analysis indicated g_1 (Michaelis-Menten constant) may have a slight temperature effect on hypocotyl length. We are unable to validate this experimentally and therefore will include it as a potential temperature dependent parameter.

Based on this analysis and our biological constraints we introduced temperature sensitivity to the following parameters: n_1 (formation rate of P_r^c), n_2 (formation rate of PIF), p_1 (dark reversion rate), m_3 (PIF- dependent degradation of Pfr), m_4 (Pfr- dependent degradation of PIF), m_5 (PIF degradation), α (basal growth rate), g_1 (Michaelis-Menten constant) and b_1 (inhibition constant of X). The exact value of Q_{10} for each parameter was calculated using simultaneous least-square fitting to 1dp to our experimental data at 27°C (Fig. 1a) and is listed in Supplementary Table 1.

Interestingly changes in the temperature dependence of Model II parameters were insufficient to recreate the fluence rate dependent increase in hypocotyl elongation at high fluence rate shown at

27°C (Fig. 3e). This supports our conclusion that dr cannot drive the switch, and the new temperature component X also cannot elicit the switch. This implies Model II is unable to recreate the temperature and fluence rate dependent rise in hypocotyl elongation in its current form. To confirm this we used LHS to assign Q_{10} values between 0-5 for each parameter then ran the model for 200,000 combinations of Q_{10} values. None of the simulated model outcomes could recreate the 27°C increase in hypocotyl elongation at high fluence rates whilst ensuring the model was consistent with both WT data at low fluence rates and phyB and PIF protein levels (Fig. 1a and Supplementary Fig. 1c, 3a). From this we can conclude that the existing components in Model II are not sufficient to elicit the photothermal switch. This led us to investigate possible methods by which a temperature dependent increase in hypocotyl elongation could be achieved through the development of Model III.

1.5 Details of Model III

Analysis of lines expressing 35S::PIF4 or 35S::PIF5 showed that while hypocotyl elongation was suppressed at higher fluence rates at 17°C, it was promoted, particularly in the 35S::PIF5 line, at 27°C (Fig. 4a,b). This provided support for the inclusion of X as a PIF suppressor at 17°C. Coupled with our data showing that PIF levels do not rise with fluence rate at 27°C (Fig. 2a, Supplementary Fig. 1c), these results also suggested the presence of a second component that activated PIF under warmer conditions. In accordance with these findings Model II was extended to include the component "Y" which activates PIFs in a temperature and fluence rate dependent manner, producing Model III. This was achieved as follows:

$$HL = \alpha + \beta_1 \frac{PIF}{g_1 + PIF} \frac{b_1}{b_1 + X} + \beta_2 \frac{PIF}{g_2 + PIF} \frac{Y}{g_3 + Y}, \quad \textbf{(13)}$$

where parameters are denoted as previously (Fig. 4d). As with component X, there are two plausible ways to incorporate a fluence response into component Y. In one, Y is dependent on phyB through the flux from Pr to Pfr conversion (Eq. 14). In the other, it is assumed that Y is independent of phytochrome, relying on a separate light input such as photosynthesis (Eq. 15). As with component X, the formation of Y will be dependent on the wavelength and fluence rate of the light used and consequently can be modelled using the value of light intensity, N_λ.

$$\frac{dY}{dt} = k_1(P_r^{\ n} + P_r^{\ s}) - m_7 Y, \tag{14}$$

$$\frac{dY}{dt} = N_\lambda - m_7 Y. \tag{15}$$

In order for Model III to emulate the photothermal switch, the concentration of Y must also be temperature dependent, i.e. the Q_{10} of m_7 is high. We estimated the unknown parameters in Eq 13 using simultaneous simulated annealing least-square fitting to all WT data. Once again we use χ^2 –values to determine the best model input for Y. The χ^2-values are 0.068 and 0.066 for phyB dependent and independent Y, respectively. Based on this method of model selection it is not possible to differentiate between the two hypotheses for Y input (Supplementary Fig. 7c, d). Purely for consistency with X, further analysis was conducted with Y as phyB-dependent. Using the Bayesian Information Criterion (BIC) we were able to directly compare Model III with the temperature dependent Model II. This analysis concluded that Model III is preferable (BIC of Model II minus the BIC of Model III is 5.78).

Strikingly, with the inclusion of Y, Model III matches the experimental data extremely well at both temperatures and at all fluence rates (Supplementary Fig. 7c, d). We then tested the robustness of Model III by establishing whether the model simulations could match experimental

mutant data. The *phyB* mutant has constitutively elongated hypocotyls across fluence rates (Supplementary Fig. 8a), while *pif* mutants have short hypocotyls and reduced fluence rate dependency (Supplementary Fig. 8b). To reproduce the null *phyB* mutant we decreased the rate of phyB synthesis to zero (Supplementary Fig. 8a). For *pif* mutants we decreased the formation rate of PIF by 25% for each *PIF* gene family knockout (e.g. PIF1, PIF3, PIF4 and PIF5) simulated. Only the *pifq* mutant (which lacks all four PIFs) is fluence rate independent. In all cases the model simulations matched the mutant data, which reinforces confidence in the model structure.

To further test the model we determined how Model III would react to light delivered continuously or in pulses. By replacing Eq. 13 in Model III with a time dependent equation for hypocotyl length (Eq. 16) we created a dynamical model of the whole system from which we were able to simulate hypocotyl length at 17°C and 27°C, under constant light compared to 5 or 15 minute pulses. The pulses delivered the same total fluence of light.

$$\frac{dHL}{dt} = \alpha + \beta_1 \frac{PIF}{g_1 + PIF} \frac{b_1}{b_1 + X} + \beta_2 \frac{PIF}{g_2 + PIF} \frac{Y}{g_3 + Y} - m_8 HL, \quad (16)$$

Interestingly, model simulations showed that pulse conditions could not elicit the temperature-dependent shift in hypocotyl length (Supplementary Fig. 8c). The model therefore predicts that the photothermal switch requires constant illumination. Indeed, we observe a qualitative match to data from plants grown under matched conditions, validating the model prediction (Supplementary Fig. 8d).

Supplementary references:

1 Nagy, F. & Schafer, E. Phytochromes control photomorphogenesis by differentially regulated, interacting signaling pathways in higher plants. *Annual review of plant biology* **53**, 329-355, doi:10.1146/annurev.arplant.53.100301.135302 (2002).

2 Casal, J. J., Luccioni, L. G., Oliverio, K. A. & Boccalandro, H. E. Light, phytochrome signalling and photomorphogenesis in Arabidopsis. *Photochemical & photobiological sciences : Official journal of the European Photochemistry Association and the European Society for Photobiology* **2**, 625-636 (2003).

3 Rausenberger, J. *et al.* An integrative model for phytochrome B mediated photomorphogenesis: from protein dynamics to physiology. *PloS one* **5**, e10721, doi:10.1371/journal.pone.0010721 (2010).

4 Al-Sady, B., Ni, W., Kircher, S., Schafer, E. & Quail, P. H. Photoactivated phytochrome induces rapid PIF3 phosphorylation prior to proteasome-mediated degradation. *Molecular cell* **23**, 439-446, doi:10.1016/j.molcel.2006.06.011 (2006).

5 Park, E. *et al.* Phytochrome B inhibits binding of phytochrome-interacting factors to their target promoters. *The Plant journal : for cell and molecular biology* **72**, 537-546, doi:10.1111/j.1365-313X.2012.05114.x (2012).

6 Jang, I. C., Henriques, R., Seo, H. S., Nagatani, A. & Chua, N. H. Arabidopsis PHYTOCHROME INTERACTING FACTOR proteins promote phytochrome B polyubiquitination by COP1 E3 ligase in the nucleus. *The Plant cell* **22**, 2370-2383, doi:10.1105/tpc.109.072520 (2010).

7 Mancinelli, A. in *Photomorphogenesis in Plants* (eds R. E. Kendrick & G. H. M. Kronenberg) Ch. 10, 211-269 (Springer Netherlands, 1994).

8 Monte, E. *et al.* The phytochrome-interacting transcription factor, PIF3, acts early, selectively, and positively in light-induced chloroplast development. *Proceedings of the National Academy of Sciences of the United States of America* **101**, 16091-16098, doi:10.1073/pnas.0407107101 (2004).

9 de Lucas, M. *et al.* A molecular framework for light and gibberellin control of cell elongation. *Nature* **451**, 480-484, doi:10.1038/nature06520 (2008).

10 Hornitschek, P., Lorrain, S., Zoete, V., Michielin, O. & Fankhauser, C. Inhibition of the shade avoidance response by formation of non-DNA binding bHLH heterodimers. *The EMBO journal* **28**, 3893-3902, doi:10.1038/emboj.2009.306 (2009).

11 Hao, Y., Oh, E., Choi, G., Liang, Z. & Wang, Z. Y. Interactions between HLH and bHLH factors modulate light-regulated plant development. *Molecular plant* **5**, 688-697, doi:10.1093/mp/sss011 (2012).

12 Schäfer, E. & Schmidt, W. Temperature dependence of phytochrome dark reactions. *Planta* **116**, 257-266, doi:10.1007/BF00390231 (1974).

13 Thomas, A., Tomos, A. D., Stoddart, J. L., Thomas, H. & Pollock, C. J. Cell expansion rate, temperature and turgor pressure in growing leaves of Lolium temulentum L. *New Phytologist* **112**, 1-5, doi:10.1111/j.1469-8137.1989.tb00301.x (1989).

14 Adam, E. *et al.* Altered dark- and photoconversion of phytochrome B mediate extreme light sensitivity and loss of photoreversibility of the phyB-401 mutant. *PloS one* **6**, e27250, doi:10.1371/journal.pone.0027250 (2011).

15 Kurosawa, G. & Iwasa, Y. Temperature compensation in circadian clock models. *Journal of theoretical biology* **233**, 453-468, doi:10.1016/j.jtbi.2004.10.012 (2005).

16 Hegarty, T. W. Temperature Coefficient (Q10), Seed Germination and Other Biological Processes. *Nature* **243**, 305-306 (1973).

17 Dixon, M., Webb, E. C., Thorne, C. J. R. & Tipton, K. F. *Enzymes* **Longman** (1979).

18 Koini, M. A. *et al.* High temperature-mediated adaptations in plant architecture require the bHLH transcription factor PIF4. *Current biology : CB* **19**, 408-413, doi:10.1016/j.cub.2009.01.046 (2009).

19 Kumar, S. V. *et al.* Transcription factor PIF4 controls the thermosensory activation of flowering. *Nature* **484**, 242-245, doi:10.1038/nature10928 (2012).

20 Foreman, J. *et al.* Light receptor action is critical for maintaining plant biomass at warm ambient temperatures. *The Plant journal : for cell and molecular biology* **65**, 441-452, doi:10.1111/j.1365-313X.2010.04434.x (2011).

21 Rockwell, N. C., Su, Y. S. & Lagarias, J. C. Phytochrome structure and signaling mechanisms. *Annual review of plant biology* **57**, 837-858, doi:10.1146/annurev.arplant.56.032604.144208 (2006).
